# Editorial: Biomaterials and biologicals for disease treatment

**DOI:** 10.3389/fbioe.2025.1572221

**Published:** 2025-04-10

**Authors:** Bo Zhi Chen, Yan Wang, Mohammad Ashfaq

**Affiliations:** ^1^ Beijing Laboratory of Biomedical Materials, College of Materials Science and Engineering, Beijing University of Chemical Technology, Beijing, China; ^2^ Department of Chemical Engineering, School of Chemical Engineering, Northwest University, Xi’an, China; ^3^ Department of Biotechnology, University Centre for Research, Development (UCRD), Chandigarh University, Mohali, Punjab, India

**Keywords:** biomaterails, biologics, diagnosis, disease treatment and therapies, biopolymers

## Introduction

Biomaterials have been investigated to diagnose and treat various diseases, mainly cancer and diabetes, cardiovascular diseases, and infectious disease management. Although most studies focus on the use of different biomaterials in the domain of the development of rapid, ultra-sensitive sensors, transdermal patches, microneedle-based delivery systems, vaccines, optometry, and agriculture (Chen et al., 2022; Drabczyk et al., 2024a; Wang B. et al., 2024; Wang R. et al., 2024). There are many reasons to choose biomaterials, which have revolutionized advanced medicine and offer exceptional opportunities for diagnosing and treating diseases (Li et al., 2021; Pablos et al., 2024). With the advancement in modern medicine, researchers try to explore newer avenues that help to interplay between materials sciences, especially nanomaterials, biological interactions, and their clinical applications to unveil the potential of biomaterials for the diagnosis and treatment of diseases (Alshangiti et al., 2023; Huang et al., 2024).

This Research Topic, “Biomaterials and Biologicals for Disease Treatment,” highlights the latest advancements in biomaterials and biologicals for treating diseases by using advanced approaches and revolutionary research addressing global health challenges. Combining biomaterials and biologicals into therapeutic approaches has ushered in a newer era of precision medicine (Lu et al., 2024). Biomaterials comprise polymers, metals, non-metals, and ceramics, engineered to interact with biological systems to augment therapeutic outcomes. Additionally, biological cells and growth factors provide functional components that must address the main cause of diseases. Indeed, such tactics have facilitated innovations in drug delivery, tissue engineering, medicine, and immunotherapy (Drabczyk et al., 2024b; Lele et al., 2024; S. et al., 2024; Sanchez Armengol et al., 2024).

Numerous studies suggested the roles of biomaterials in numerous end applications. For example, Ertas et al. focused on the role of biomaterials in diagnosing and treating coronavirus. Biomaterials can effectively contribute to developing newer vaccines, drug delivery, and new therapeutic agents, which can be used to treat coronavirus (Ertas et al., 2021). Another study focuses on the use of biomaterials in dry eyes; with the help of biomaterials, the therapeutic efficacy of drugs significantly increases, which helps to control dry eye diseases. Although only a few studies have been published on treating dry eye diseases, it is difficult to compare the role of different biomaterials in dry eye disease (Thacker et al., 2023). Additionally, long-term studies would be required to evaluate ocular toxicity and biocompatibility, which might enhance their transition to human use (Thacker et al., 2023). Likewise, another group focuses on the different classes of biomaterials, mainly microneedle-based delivery systems for treating insulin delivery, anti-aging properties, cancer, wound healing, sensors, and agriculture. Microneedle-based technology offers a newer avenue for the treatment and diagnosis of diseases, as its painless delivery system (Wang et al., 2016; Jin et al., 2018; Ganeson et al., 2023; Hu et al., 2024; Kumari et al., 2024). Another approach is polymer-bioconjugates, as conjugation of polymers with numerous biomolecules including growth hormones, proteins, DNA, and RNA provides next-generation tools, which are easily achieve therapeutic efficacy through the delivery of biomolecules (Chen et al., 2020; Barman et al., 2023; Sun et al., 2023).

Indeed, there are many more examples of biomaterials and their applicability to treating diseases. As we assume the next-generation prospect, it’s obvious that biomaterials will continue to play a decisive role in disease management. Future research integrating sensing capabilities and artificial intelligence integration will allow for real-time biomaterial changes to better address the treatment of diseases. Additionally, to increase the material’s longevity as well, this may also incorporate materials that can self-heal. As researchers continue to innovate with biomaterials, the distribution of clinical trials will shift to focus on these future biomaterials and their clinical applications. Increases in composites and combinations, autologous materials, and bioactive and bioresorbable materials are anticipated due to the development of these advanced biomaterials (Lele et al., 2024). This issue includes understanding the current research of biomaterials on treating various diseases, discussing imminent therapeutic implications, and fostering a deeper understanding of researchers for future research and clinical application. For instance, Ali et al. focus on synthesizing laboratory-prepared transparent 2-hydroxyethyl methacrylate-based Fresnel lenses for ocular management (Ali et al.). Zhou et al. focus on the multi-model triggered release nanocarriers for the treatment of colon cancer. Rayat Pisheh et al. focus on the amniotic membrane as a biological scaffold for treating cardiovascular diseases. Another study focuses on nanomaterials for the photothermal treatment of myocardial infarction (Yang et al.). Indeed, these studies significantly improved the understanding the biomaterials and biologics for the treatment of diseases.

## Conclusion

Biomaterials have transformed advanced medicine, providing exceptional opportunities for diagnosing and treating diseases. This editorial reveals how biomaterials and biologicals manage the diagnosis and treatment of diseases. Understanding the roles of biomaterials and biologicals is paramount to managing diseases. We believe that this Research Topic might provide newer avenues to understand biomaterials’ role, which can translate into clinical practices.
